# Cardiac herniation identified without any symptoms following extrapleural pneumonectomy: a case report

**DOI:** 10.1186/s44215-025-00197-3

**Published:** 2025-03-11

**Authors:** Ryosuke Tokuda, Satoshi Ikebe, Masayoshi Inoue

**Affiliations:** 1 Department of General Thoracic Surgery, Fukuchiyama City-Hospital, 231 Atsunaka-Machi, Fukuchiyama, 620-8505 Japan; 2 Division of Thoracic Surgery, Department of Surgery, Graduate School of Medical Science, Kyoto Prefectural University of Medicine, 465 Kajii-Cho, Kamigyo-Ku, Kyoto, 602-8566 Japan

**Keywords:** Asymptomatic cardiac herniation, Extrapleural pneumonectomy, Diffuse pleural mesothelioma

## Abstract

**Background:**

Cardiac herniation, especially right-sided herniation, is a fatal complication which causes sudden hypotension due to obstruction of the vena cava. Here, we describe a case of cardiac herniation identified without any symptoms after right extrapleural pneumonectomy performed for diffuse pleural mesothelioma.

**Case presentation:**

A 72-year-old man with diffuse pleural mesothelioma underwent a right extrapleural pneumonectomy after chemotherapy. The tumor had widely invaded the pericardium, necessitating pericardial resection. The pericardial defect was approximately 10 × 6 cm and was reconstructed with a 0.1-mm polytetrafluoroethylene sheet. Routine chest radiographs taken just after the operation were normal. A chest radiograph on postoperative day one revealed cardiac herniation but he remained hemodynamically stable. An urgent re-thoracotomy was performed for pericardial reconstruction. Severe hypotension occurred immediately before the operation, but was improved upon placing the patient in the left lateral decubitus position. Postoperatively, he developed postoperative complications including chylothorax and empyema, and was discharged 118 days after surgery.

**Conclusions:**

Cardiac herniation can occur without any symptoms following right pneumonectomy with pericardiectomy. Urgent reoperation is warranted due to the high risk of impending shock, even in hemodynamically stable patients.

**Supplementary Information:**

The online version contains supplementary material available at 10.1186/s44215-025-00197-3.

## Background

Cardiac herniation after extrapleural pneumonectomy (EPP), especially right-sided herniation, is a life-threatening complication due to obstruction of the vena cava and resulting hypotension [1]. Herein, we describe a case of cardiac herniation identified without any symptoms after right EPP for diffuse pleural mesothelioma (DPM).

## Case presentation

**Fig. 1:**
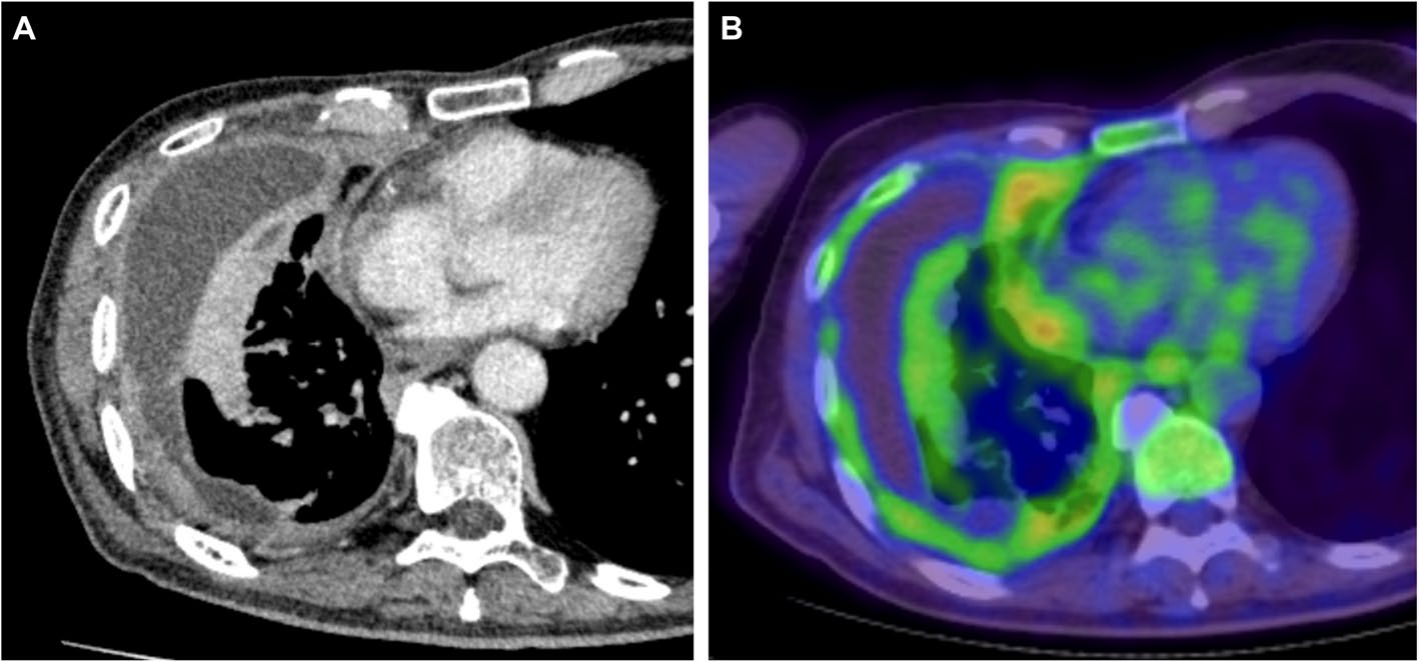
Preoperative chest computed tomography before chemotherapy. **A** Contrast-enhanced chest computed tomography reveals right pleural effusion with thickening of the pleura and **B** positron emission tomography computed tomography shows F-fluorodeoxyglucose uptake in the pleural

A 72-year-old man with performance status 0 and no comorbidities was referred to our hospital with a chief complaint of exertional dyspnea. Chest computed tomography (CT) showed a right-sided pleural effusion with pleural thickening (Fig. 1A), and positron emission tomography CT showed F-fluorodeoxyglucose uptake in the pleural (Fig. 1B).

**Fig. 2:**
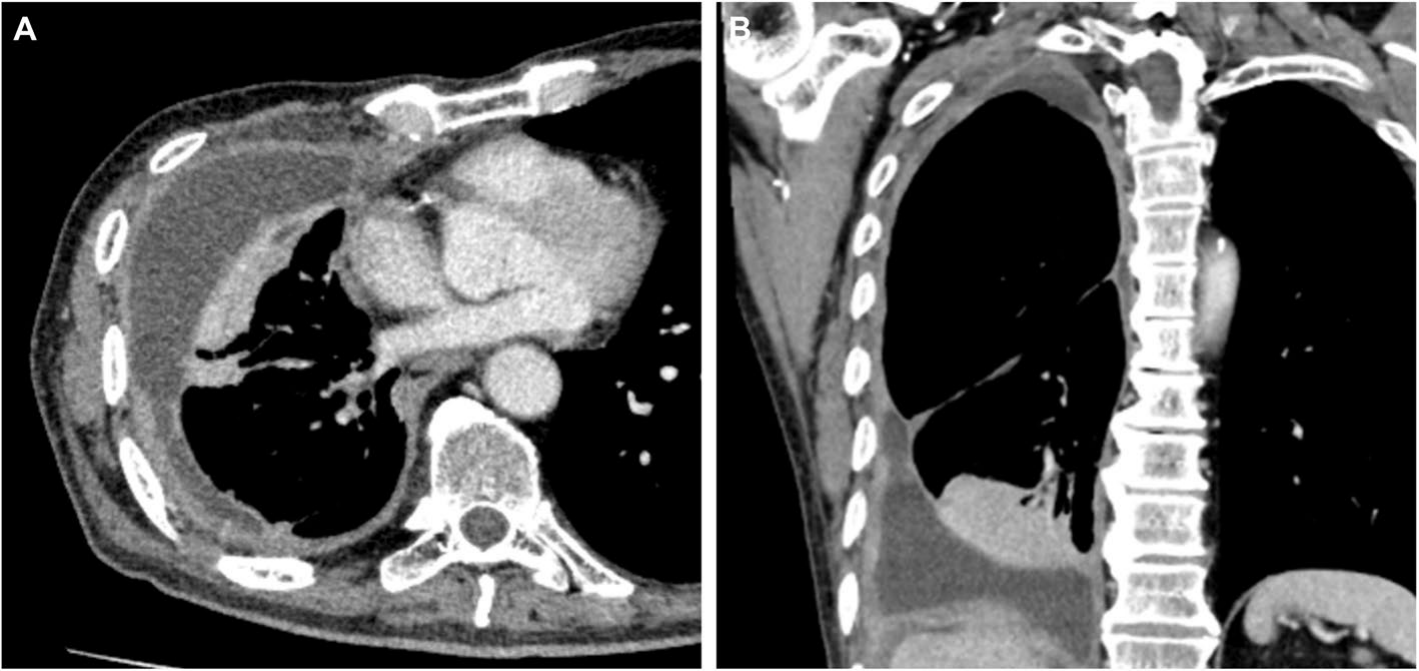
Preoperative chest computed tomography after chemotherapy. **A**, **B** Contrast-enhanced chest computed tomography reveals right pleural effusion with thickening of the pleura. Tumor invasion into the lung, diaphragm, and pericardium is suspected

A thoracoscopic pleural biopsy revealed epithelial-type DPM. The patient was diagnosed as cT3 (pericardium) N0M0 stage IB. After three courses of chemotherapy (cisplatin and pemetrexed), we re-evaluated the chest CT scan (Fig. 2) and assessed the disease to be stable according to the Response Evaluation Criteria in Solid Tumors. After chemotherapy, the patient was diagnosed as ycT3 (pericardium) N0M0 stage IB and resectable after consensus at institutional cancer board, which included thoracic surgeons, medical oncologists, pulmonologists, and radiologists. The patient was supposed to require EPP not pleurectomy decortication, because the tumor was suspected to have invaded the right lung parenchyma with partial atelectasis of the right lower lobe. The respiratory function test could not be adequately evaluated due to the patient’s inability to follow the instructions. However, considering the patient’s ability to climb five floors in a stair-climbing test and the normal findings on the echocardiography (ejection fraction 75%, no valvular disease, tricuspid regurgitation pressure gradient 19 mmHg), the patient was considered to have sufficient functional capacity for EPP.

**Fig. 3:**
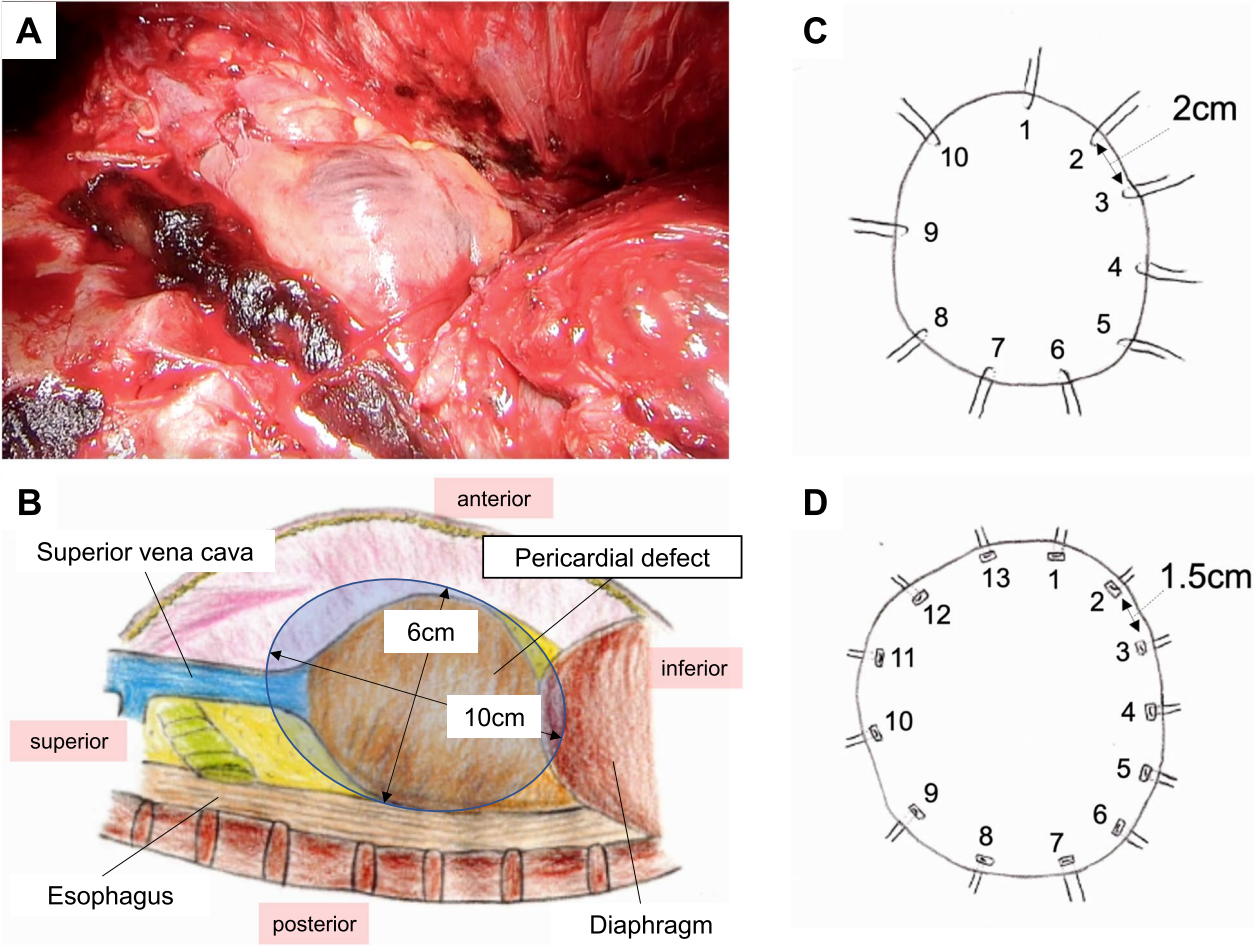
Extent of pericardial defects and methods for pericardial reconstruction. **A**, **B** Intraoperative findings on the extent of pericardial defects during initial surgery, which was approximately 10 × 6 cm. **C** During the initial operation, the pericardium is reconstructed using 2–0 non-absorbable monofilament sutures with 10 interrupted stitches, spaced approximately 2 cm apart. **D** During reoperation, the pericardium is reconstructed using 2–0 non-absorbable monofilament sutures with 13 horizontal mattress sutures, spaced approximately 1.5 cm apart

**Fig. 4:**
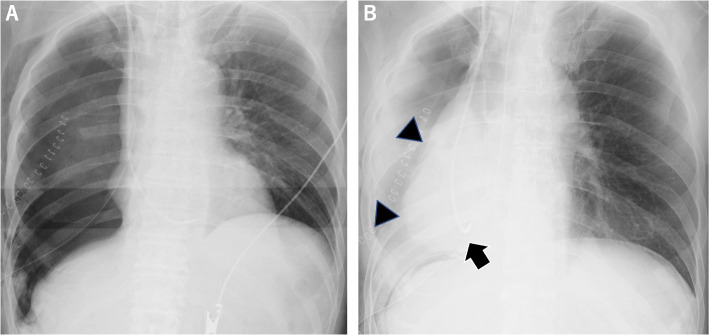
Postoperative chest radiograph. **A** A routine chest radiograph taken in the operating room has unremarkable findings. **B** A chest radiograph taken postoperative day one reveals a shift of the heart to the right thoracic cavity (arrowhead) with an abnormal positioning of the Swan-Ganz catheter (arrow)

**Fig. 5:**
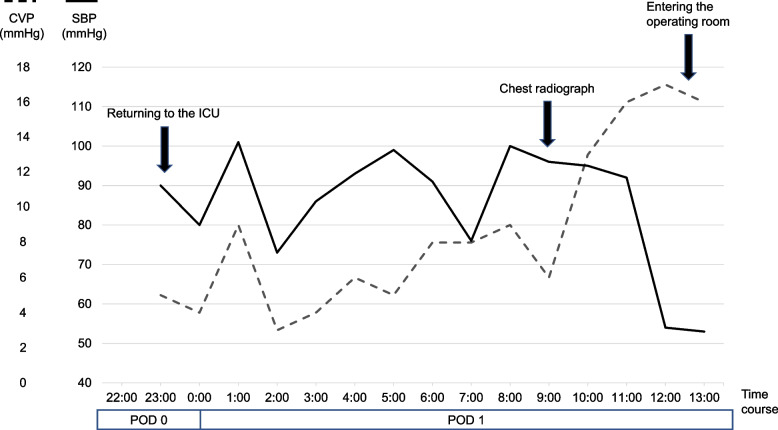
Perioperative clinical time course at right extrapleural pneumonectomy. In the intensive care unit, the patient remained hemodynamically stable, though routine chest radiograph diagnosed cardiac herniation. The patient developed marked hypotension and elevated central venous pressure immediately before entering the operating room

During the surgery, the tumor had invaded the pericardium and diaphragm, requiring extensive resection and reconstruction. The pericardial defect was approximately 10 × 6 cm (Fig. 3A, B) and was reconstructed with a 0.1-mm polytetrafluoroethylene (PTFE) sheet using 2–0 non-absorbable monofilament sutures with 10 interrupted stitches (Fig. 3C). A chest drain was placed to water seal without suction. Routine chest radiographs performed in the operating room showed unremarkable findings (Fig. 4A). The patient returned to the intensive care unit and mechanically ventilated (assist control, pressure control ventilation, positive end-expiratory pressure 5 cmH2O, inspiratory pressure 15 cmH2O) on the day of the procedure. Though the patient’s vital signs were stable, a chest radiograph taken on postoperative day one revealed a shift of the heart to the right thoracic cavity (Fig. 4B). We clamped the chest drain and decided to immediately perform a re-thoracotomy based on the diagnosis of postoperative cardiac herniation. Before undergoing a re-thoracotomy, he developed sudden hypotension and central venous pressure elevation 3 h after the cardiac herniation diagnosis (Fig. 5). His blood pressure improved when he was placed in the left lateral decubitus position. Intraoperatively, the heart was anatomically repositioned due to the lateral decubitus position. The sutures fixing the PTFE sheet had dislodged, with subsequent rupture of the pericardial remnant posteriorly and inferiorly. There was no surrounding connective tissue of the heart at all (Additional file). To reconstruct the pericardium, we used a 0.1-mm PTFE sheet and 13 horizontal mattress sutures using 2–0 non-absorbable monofilament sutures with felt pledgets, and the pitch was shortened from that in the initial procedure (Fig. 3D). He developed postoperative complications of chylothorax and empyema, and was discharged 118 days postoperatively. The patient had tumor recurrence in the contralateral lung and subsequently died 6 months post-surgery.

## Discussion

Cardiac herniation is a rare but fatal complication, with a 100% mortality rate in undiagnosed cases [2]. Risk factors for cardiac herniation include increased intrathoracic pressure associated with mechanical ventilator support or coughing; chest drainage on the surgical side; preoperative chemotherapy; positional changes; and large-sized pericardial defects, especially on the right side [3, 4].

**Fig. 6:**
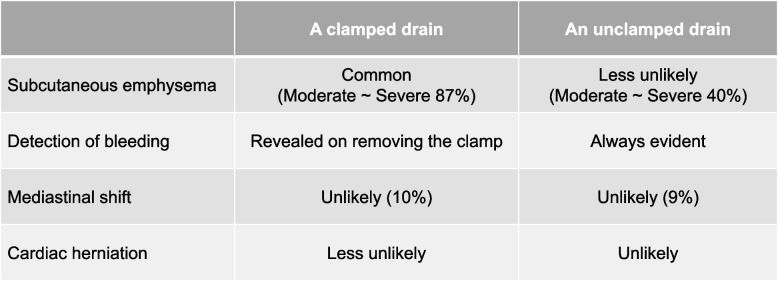
Pros and cons of the clamping or unclamping the drain after pneumonectomy. Unclamping the drain results in less subcutaneous emphysema and better detection of postoperative bleeding; however, the risk of cardiac herniation is increased

In the present case, postoperative ventilation, thoracic drainage with a water seal, preoperative chemotherapy, and a large pericardial defect likely induced right cardiac herniation. Cardiac herniation occurs due to pressure differences between the two hemithoraces, often caused by ventilation [3] or coughing [5, 6]. In this case, ventilation caused hyperinflation of the remaining lung and increased intrathoracic pressure, which led to a mediastinal shift toward the affected side. There is insufficient evidence to determine whether clamping or unclamping a drain with a water seal is ideal following EPP; therefore, the decision is made empirically by surgeons [7] according to the advantages and disadvantages of each strategy based on previous studies [3, 8] (Fig. 6). Patients with the risk for cardiac herniation, as in this case, should have been managed with a clamped drain. Chemotherapy is believed to contribute to fibrotic changes in the pericardium and the weakening of the pericardium [4]. Cardiac herniation occurs in 3.2% (2/63) of patients following EPP with preoperative chemotherapy for DPM [9], while only one out of 328 patients following EPP without preoperative chemotherapy for DPM developed cardiac herniation, which led to death [10]. Preoperative chemotherapy may have weakened the pericardium and contributed to the development of cardiac herniation in this case as well. Technically, there was room for improvement in addressing the large pericardial defect, especially in the initial surgery. During the initial surgery, the pericardial defect was reconstructed with 10 interrupted stitches; however, it was insufficient to hold the reconstructed pericardium against the increased pressure caused by the large defects on the pericardial patch and sutured tissue. During reoperation, we reconstructed the pericardium with a shorter pitch (approximately from 2 to 1.5 cm) and horizontal mattress sutures with felt pledget to distribute the pressure; continuous sutures are also performed in other institutions [3].

Right cardiac herniation can occur asymptomatically. The mechanism of cardiac herniation differs between the right and left sides. On the right side, the entire heart protrudes into the right thoracic cavity through the pericardial defect, and cardiac herniation leads to obstructive shock due to anti-clockwise rotation around the superior and inferior vena cavae in CT view. Thus, it is more likely to occur when the pericardial defect is large. On the left side, the ventricular apex protrudes and is constricted by the pericardium, and cardiac herniation can lead to myocardial infarction [1]. In the present case, the diagnosis of cardiac herniation was made based on a routine chest radiograph on postoperative day one, and the patient did not demonstrate hemodynamic instability at the time of diagnosis. There have been four reported cases of cardiac herniation without hemodynamic collapse [5, 11–13], all of which occurred on the right side, interestingly. Right cardiac herniation occurs in patients with large pericardial defects and can lead to sudden hypotension [3]. The position of the prolapsed heart can change depending on the patient's position and intrathoracic pressure. Cases with minimal dislocation of the heart may not result in hemodynamic deterioration, as shown in the present case. However, sudden deterioration and shock occurred subsequently. When cardiac herniation is suspected on chest radiograph, emergency surgery should be performed immediately, even if the patient is currently hemodynamically stable.

Cardiac herniation without any symptoms detected on chest radiography is associated with impending hemodynamic shock and necessitates urgent pericardial repair.

## Supplementary Information

Additional file 1: Re-thoracotmy for repairing the pericardium. Re-exploring the chest revealed cardiac herniation with the rupture of the posterior and inferior pericardial remnant. We reconstructed the pericardium using a 0.1 mm PTFE sheet and 13 horizontal mattress sutures with 2–0 non-absorbable monofilament sutures with felt pledgets.

## Data Availability

For more information, please contact the journal or corresponding author.

## Abbreviations

CT: Computed tomography
DPM: Diffuse pleural mesothelioma
EPP: Extrapleural pneumonectomy
PTFE: Polytetrafluoroethylene
